# Radon level in a Nigerian University Campus

**DOI:** 10.1186/s13104-015-1447-7

**Published:** 2015-11-14

**Authors:** Olusegun Temitope Afolabi, Deborah Tolulope Esan, Bosun Banjoko, Benjamin A. Fajewonyomi, James Enajero Tobih, Babakayode Babajide Olubodun

**Affiliations:** Department of Community Health, College of Health Sciences, Obafemi Awolowo University, Ile-Ife, Nigeria; Department of Chemical Pathology, College of Health Sciences, Obafemi Awolowo University, Ile-Ife, Nigeria; Depatment of E.N.T., Ladoke Akintola University Teaching Hospital, Osogbo, Nigeria; Department of Geology, Obafemi Awolowo University, Ile-Ife, Nigeria

**Keywords:** Radon, Carcinogen, Environment, Indoor air quality, University

## Abstract

**Background:**

Globally, radon is a natural contaminant that affects indoor air quality. Several epidemiological studies have implicated high radon levels in the causality of lung cancer. The study therefore determined the environmental level of radon in selective offices in the Obafemi Awolowo University, Ile-Ife. The study employed a descriptive cross-sectional design. A Pro 3-series radon detector was used to determine the radon levels in randomly selected offices. The instrument was set-up in each office and after 48 h, reading was taken and recorded on a proforma data sheet. The structural characteristics of the offices were also assessed by observation.

**Results:**

The result revealed that the radon level obtained in the sampled offices ranged from 0.0 to 5.3 pCi/L (196 Bq/m^3^). The median concentration of radon obtained from sampled offices was 0.9 pCi/L. Almost all (95 %) of the offices had radon levels within the ‘permissible’ reference level recommended by World Health Organization. Radon levels also showed a statistically significant decline with height of office building with the mean concentration of radon in offices located on the basement, ground floor and first floor being 1.54 ± 1.32, 0.99 ± 0.56, 0.63 ± 0.41 pCi/L respectively, (F statistic 5.8, p < 0.001).

**Conclusion:**

The radon levels obtained in most assessed offices in Obafemi Awolowo University were found to be within the permissible reference levels. Mitigation measures should be put in place in the few offices above permissible levels.

## Background

Radon exposure is one of the leading causes of death from lung cancer in the United States each year, with thousands of individuals succumbing to its effects [1]. Also in the United Kingdom (UK), breathing in radon is the second largest cause of lung cancer resulting in up to 2000 fatal cancers per year [2].

Radon (more properly known as radon-222) comes from uranium which occurs naturally in many rocks and soils. Radon is a naturally occurring radioactive gas that is colourless, tasteless and odourless. This gas contaminates indoor air from soil and rocks by molecular diffusion governed by Fick’s law, or gaseous diffusion described by Darcy’s law or a combination of both mechanisms and thereby infiltrate housing foundations [3]. The highest levels are usually found in underground spaces such as basements, caves and mines. High concentrations are also found in ground floor buildings [4]. Higher radiation levels are associated with igneous rocks such as granite, tuff, while lower levels are found with sedimentary rocks [5]. The radioactive gas radon is a potential hazard in many homes and workplaces. It can seep out of the ground and build up in houses and indoor work places.

Radon exposure occurs when people come in contact with the radioactive radon particles that have been caused from decay of uranium and radium in the ground. It is a product of ^226^Ra decay in the ^238^U series. There are no immediate symptoms from exposure to radon, making it difficult to know if there is a problem until sometimes later when it is already at a toxic level [1]. Therefore, the attending physician must have a high index of suspicion. Radon is also present outdoors but in extremely low levels that are not harmful to humans, but when it gets into poorly ventilated areas such as a basement or an enclosure, it builds up to dangerous levels [1]. Radon exposure in homes/offices may arise from certain subsurface rock formations and also from certain building materials (e.g. granites); greatest risk of radon exposure are from tight, insufficiently ventilated buildings and buildings that have leaks that let in soil air from the ground into basement and dwelling rooms. Trouble starts when radon enters a home through cracks in the foundation, floors and walls and builds up to a toxic level which can manifest clinically. The radon levels can rise to the point where they have an impact on a person’s health, especially if the home is well insulated thermally. The fact that radon comes from the ground means, the closer the level of the house to the ground, the more the danger of radon exposure [1].

Radon is known to decay quickly with a half life of 3.82 days by alpha emission causing significant damage to the sensitive cells in the lungs. Radon’s primary hazard arises from inhalation of the gas and its highly radioactive heavy metallic decay products (Polonium, Lead and Bismuth) which tend to collect on dust in the air. When these radioactive bits are inhaled, they settle in the person’s lungs and begin to do damage to the mucosa linings [6]. Exposure to radon over a long time can result in enough damage to the pulmonary mucosa leading to cancer.

Over 50 % of the average individual radiation dose comes from exposure to radon decay products. Two of the radon decay products, Polonium-218 and Polonium-214, account for the majority of the radiation exposure to lungs. If inhaled, radon decay products (polonium-218 and polonium-214, solid form), unattached or attached to the surface of aerosols, dusts, and smoke particles, become deeply lodged or trapped in the lungs, where they can radiate and penetrate the cells of mucous membranes, bronchi, and other pulmonary tissues [6]. The ionizing radiation energy affecting the bronchial epithelial cells is believed to initiate the process of carcinogenesis. Although radon-related lung cancers are mainly seen in the upper airways, radon increases the incidence of all histological types of lung cancer, including small cell carcinoma, adenocarcinoma, and squamous cell carcinoma. Lung cancer due to inhalation of radon decay products constitutes the only documented risk associated with radon. In studies done on miners, variables such as age, duration of exposure, time since initiation of exposure and especially the use of tobacco have been found to influence individual risk. In fact, the use of tobacco multiplies the risk of radon-induced lung cancer enormously [7].

Because we are building homes without radon resistant features, more people are exposed to radon than ever before [8]. Residential radon exposure is based on a combination of how long an individual is exposed and the radon level present in the home [9]^.^ Uranium and Radium in the soil is considered to be the main source of indoor radon concentration, although building materials especially quartz and cement can make a significant contribution to the level of natural radioactivity in closed places [10].Radon concentrations in dwellings depend on meteorological and geological conditions, construction materials and ventilation [11].

However, this cannot be detected except by testing. It has therefore become necessary to investigate work environment for elevated radon levels. As far as the published scientific literature is concerned, only one study has been conducted on the threat radon poses to public health in houses in Nigeria [12], even though a study on the radon soil gas has been conducted in Ile-Ife [13]. The interest in radon became urgent because of the known interest in cancer which has heightened in recent years. The United States of America and other developed countries have identified the health risk this gas poses to humans and are developing mitigating measures to keep exposure levels low; unfortunately the story is different in many developing countries like Nigeria. Radon is only known to few people, and there is limited documented research yet on its health hazards in Nigeria. A thorough search of the literature suggests that Nigeria has few records of its radon emanation levels either at the level of home or at work places.

It cannot be over-emphasized that radon is harmful at higher levels and that many lung cancers could be initiated by radon exposures [7]. There is an urgent need to, therefore, assess the vulnerability of people who live in areas that may have high levels of radon concentration such as residential areas and workplaces located in mountainous terrains such as the Obafemi Awolowo University (OAU) campus.

A mountainous terrain like the study area where structures have been built according to the landscape may be a source of high radon emissions due to the underlying rock formations on which the buildings have been sited. In view of potential health risks that people living in the study area may be subjected to unknowingly due to radon emanation from subsoil into buildings/office spaces, the study therefore assessed the environmental level of radon in selected offices of the OAU and also assessed the physical dimension of office spaces in relation to radon emanation levels.

## Methods

The study was conducted in various office buildings of the Obafemi Awolowo University, Ife, Osun State. Obafemi Awolowo University (O.A.U) is a comprehensive public institution established in 1962 as the University of Ife. The landscape is marked by many steeply inclining hills of granite rock formation- the inselbergs- whose slopes are covered with dense vegetation, forming a natural green back drop to the campus. Its topography is hilly and there are many steep slopes, ranging from a 6 to 12 % incline. The University campus is divided into 3 major zones; academic, student residential area and staff quarters. The academic zone consisting of the main core and its extensions contains the 13 faculties and Departmental buildings, including lecture rooms, seminar rooms, libraries, laboratories, auditorium and offices. This area is located on a gently sloping area in the center of the campus designed as foreground to the nearby hills and planned as the heart of the entire university complex. Most of these building were built according to the terrain/topography which suggests a possibility of radon emanation through these grounds into the living spaces/offices in the environment.

This study employed a cross-sectional study design and the offices in the academic area and their occupants were the study population. A sample size of 87 was calculated using the Fisher’s formula with level of confidence set at 95 %; a precision of 0.05 and prevalence of attribute at 6 % which represented the proportion of households with radon levels exceeding 4 pCi/L in the U.S [14].

The buildings were stratified by underlying geology based on the classification by Adepelumi and co- researchers [13] into granite gneiss; grey gneiss and mica schist with most of the buildings in the academic area falling within the grey gneiss zone. The buildings were sampled randomly in each unit with a total of 8 buildings selected and these were further stratified into floor levels (basement, first and second) with equal sampling from the floor levels. Therefore, in each building, an average of 11 offices was selected distributed equally by floor. A total of 62 offices participated in the study yielding a non-response rate of 13 %.

The respondents were given explanation about the study and their written consent sought and obtained. Thereafter, a radon meter (Pro Series3 Radon Detector device) was used to determine radon concentrations in each office. The radon monitor was set up at about 1 m from the windows, doors, or any other potential openings in the exterior wall. The location of the instruments was placed at least 0.3 m from the exterior wall and at least 0.5 m from the floor. It was ensured that no other objects were placed within 0.1–0.2 m of the instrument. The radon meter was read after 48 h and recorded on proforma data sheet. The Radon Detector (Pro Series 3 Radon Detector Device) measures radon in picocuries per liter (pCi/L). The radon detector was pre-calibrated to measure radon activity between 0.0 and 999.9 pCi/L. However, the precision/reliability of the device was done/checked by setting up 2 devices, in the same mode and at the end of 48 h the readings were checked. It was found that the two instruments produced the same result (0.5 pCi/L). The sensor uses ionization chamber technology.

Radon levels were classified based on WHO, 2009 handbook on indoor radon [15] into permissible level (<2.7 pCi/L); risky level (2.7–4.0 pCi/L) and critical threshold level (>4 pCi/L).

During the course of data collection, dimensions of the offices were taken with tape rule and recorded; other notable observation like types of flooring, cracks, tiles and concrete were also noted. Also questionnaires were administered to occupants of sampled offices to assess their socio-demographic characteristics. Data was entered using Epidata and then exported to SPSS version 16 where analysis was done at univariate and bivariate levels. Data were presented as tables and charts with significant p value set at <0.05. Ethical clearance was obtained from the ethical review board of the Obafemi Awolowo University Teaching Hospital Complex, Ile-Ife.

## Results and discussion

### Results

Table 1 revealed the socio-demographic characteristics of occupants of sampled offices. The mean age of the sampled respondents was 43 years, the mean number of years spent in the office was 6 years and the mean length/hours of stay per day was 7.3 h. Of the 62 sampled offices, 95 % had radon levels below the permissible level while 3.2 % had radon levels at risky level and 1.6 % had above the critical threshold level with maximum record radon level being 5.3 pCi/L. The median value was 0.9 pCi/L ranging from 0.0 to 5.3 pCi/L (Table 2).

**Table 1:** Socio-demographic characteristics of occupants of sampled offices

Variable	Frequency (76)	Percent (%)
Age (years)		
21–30	9	11.8
31–40	26	34.2
41–50	28	36.8
51–60	10	13.2
61–70	3	3.9
Mean age (SD)	43.4 (9.4) years	
Number of years of office occupancy		
<1	11	14.5
Between 1–9	48	63.2
Between 10–20	14	18.4
>20	3	3.9
Mean year (SD)	5.9 (5.4) years	
Average length of stay in the office/day		
Mean (SD)	7.3 (2.5) h	
Minimum stay	2 h	
Maximum stay	12 h

**Table 2:** Radon levels obtained from sampled offices in picocuries per liter (pCi/L)

Radon level/concentration(pCi/L)	Number of offices	%
≤2.7^a^ (permissible level)	59	95.1
>2.7^b^ (risky level)	2	3.2
≥4^c^ (critical threshold)	1	1.6
Total	62	100
Median	0.9 pCi/L	

Minimum value: 0.0 pCi/L, maximum value: 5.3 pCi/L

^a^Permissible level: this is the indoor level recommended by WHO as a standard for countries to adopt

^b^Risky level: radon values at this level pose some degree of risk

^c^Critical threshold: this is dangerous level in which immediate action must be taken. Also known as the ‘Action level’

Table 3 revealed that, the basement stratum has 85.7 % of its offices having Radon levels that fall within the permissible level (<2.7 pCi/L) while 7.1 % of the offices in this stratum falls within the risky level, and 7.1 % in the critical threshold. Also, the ground floor stratum has 96 % of its radon level within the permissible level and about 4 % within the risky level whereas in the first floor stratum, all the sampled offices(100 %) falls within the permissible level.

**Table 3:** Office location and distribution of radon level

Office location	Permissible level N (%)	Risky level N (%)	Critical level N (%)	Total
Basement	12 (85.7)	1 (7.1)	1 (7.1)	14
Ground floor	27 (96.4)	1 (3.5)	0 (0.0)	28
First floor	20 (100)	0 (0.0)	0 (0.0)	20

Table 4 revealed the result of one-way analysis of variance that was done to compare the mean of the dependent variable (radon level) and the independent variable (office location-basement floor, ground floor and first floor). There was a significant difference in the means of Radon levels obtained from these 3 different strata with mean levels at the basement, ground and first floors being 1.5 pCi/L; 0.99 pCi/L and 0.66 pCi/L respectively (p < 0.001).

**Table 4:** Relationship between office location and radon levels

	Basement	Ground floor	First floor	F	P
Mean ± SD	1.54 ± 1.318	0.99 ± 0.556	0.63 ± 0.409	5.77	<0.001

Table 5 revealed that, 51.6 % of the offices were tiled, 27.4 % had rug on the floor, 12.9 % were bare concrete floor and 8.1 % of the offices were found to have cracks.

**Table 5:** Physical characteristics of sampled office space

Office characteristics	Frequency (F)	(%)
With floor tiles	32	51.6
With rug	17	27.4
With concrete floor	8	12.9
Presence of cracks	5	8.1
Total	62	100

Table 6 revealed the physical dimension of sampled office spaces. The mean surface area of the 62 offices sampled was 48.91 m^2^ (18.23), and mean area of windows was 5.72 m^2^ (2.77). The area of the room was calculated using the formular-2(length*width* + length*height + width*height). The correlation coefficient (r) = 0.1911 obtained by correlating radon level and area of office space revealed that there is no significant relationship between area of office and radon concentration level and also the r (0.0803) obtained by correlating radon level with area of windows shows that there is no relationship between area of window and radon concentration levels. Results are presented in Table 7.

**Table 6:** Physical dimension of sampled office spaces

Dimension	Area of office(N = 62)			
	Mean (m^2^)	SD	Min (m^2^)	Max (m^2^)
Room	48.91	18.23	13.9	96.7
Window	5.72	2.77	0.58	14.21

**Table 7:** Association between physical dimensions of office spaces and radon levels

	Correlation(r)	Remarks
Radon level and surface area of office	0.19	Weak correlation (p = 0.15)
Radon level and surface area of window	0.08	No relationship (p = 0.55)

Critical value at n = 62 is 0.27

**Fig. 1:**
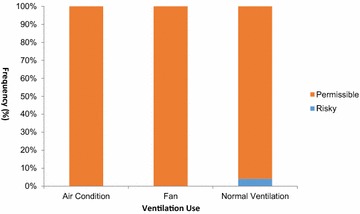
Relationship between radon level and ventilation use

In this study, 100 % of office residents that use Air conditioners had their radon level fall within the permissible level, the same with those who use fans while those who use natural ventilation had 96.8 % of their radon level within the permissible level and 3.2 % within the risky level (Fig. 1).

### Discussion

Radon Concentration obtained from sampled offices within OAU campus ranged from 0.0 to 5.3 pCi/L with the mean of 1.0 pCi/L (37 Bq/m^3^). Most of the sampled offices (95 %) fell within the ‘permissible reference level’ recommended by WHO as a standard for countries to adopt. The mean value of radon obtained in this study location is similar to the average indoor radon level estimated worldwide (about 1.3 pCi/L) [13]. The risk of radon exposure obtained in this study area can be said to be insignificant, however the US EPA believes that any radon exposure carries some degree of risk—no level of radon is safe, this is also corroborated by the study reported by Darby et al. [7]. From the mean obtained from this study, two (2) out of a thousand population could develop lung cancer for non-smokers and twenty (20) people out of a thousand could develop lung cancers among smokers [14].

Also the mean values obtained at different levels of office locations (basement 1.54 pCi/L, ground floor 0.99 pCi/L, first floor 0.63 pCi/L) differ significantly from one another. This result shows a decreasing trend of radon concentration with height probably due to increased air flushing and ventilation. This is consistent with literature which reveal that the higher the elevation in a building, the lower the radon level [4] and that higher concentrations of radon are present in basement and ground floor buildings. The reason for this is not farfetched, since radon emanation operates through the process of diffusion, the farther from the contact source the lower the concentration of radon i.e. the higher you go the lesser the concentration of radon.

To date, several field studies measuring radon and/or its decay products have been conducted. The result obtained in this study is somewhat similar to that reported by Obed et al. [12] in a university community in Ibadan a contiguous city to Ile-Ife but a bit higher than what was obtained in one of the studies done in a Ghanaian modern sandcrete building where the mean concentration was about 0.1 pCi/L. The mean values obtained at different floors also differed [16]. Also, in a study of 21 houses in the New York city region, the geometric mean of radon levels was 1.7 pCi/L in cellars and 0.83 pCi/L in first floors. The cellar values ranged from about 0.5 pCi/L up to 4.4 pCi/L. The first floor values ranged from just above outdoor background (0.25 pCi/L) up to 3.1 pCi/L. Studies of other “normal” areas of Florida and Colorado have yielded similar results [16].

Also, a maximum value of 5.3 pCi/L was obtained in one of the offices in the basement floor of the Faculty of Health Sciences. This falls under the critical threshold value of US EPA and indicates that action must be taken. EPA recommends that for values above 4 pCi/L observed in a short term test, a repeated long term test must be done and if levels are found still to be high, mitigation measures should be carried out. In European pooling study [7], the risk of lung cancer was 20 % higher for those individuals who live in houses with measured radon concentration of 2.7–5.4 pCi/L (100–199 Bq/m^3^) when compared to those with measured radon concentrations below 2.7 pCi/L (100 Bq/m^3^).

Analysis of the relationship between radon level and surface area of office was found to have a weak relationship. This is evident from the r = 0.1911 at p > 0.05. Also, correlating window dimensions and radon concentration level of sampled offices revealed no relationship (r = 0.0803 at p = 0.05). The result of this finding is similar to that obtained in a study of radon surveys in the Hong Kong Area where the mean radon concentration in office building had no correlation with the room size, floor level in office building, age of building [17].

### Conclusion

Based on this study, the siting of the University of Ife (OAU) among rocks, hale and thick forest poses mostly insignificant radon risk. An extended study however needs to be conducted in offices that are in basements. This study poses this question, how widespread is the risk of radon in Nigeria? To answer this question, it is recommended that more studies should be undertaken especially in habitation underlain by rocks so as to understand how widespread the risk of radon is in Nigeria. As far as it is known, Environmental Impact Assessment (EIA) undertaken for housing projects in this country do not yet include radon studies. Unless more studies are undertaken to understand how widespread the risk of radon is, the threat from radon exposure may remain an undisclosed health hazards for a long time to come.

## Abbreviations

EIA: Environmental Impact Assessment
EPA: Environmental Protection Agency
OAU: Obafem Awolowo University
SPSS: Statistical Programme for Social Sciences
UK: United Kingdom
US: United States of America
WHO: World Health Organization
